# Converging multi-modality datasets to build efficient drug repositioning pipelines against Alzheimer’s disease and related dementias

**DOI:** 10.1515/mr-2021-0017

**Published:** 2022-02-14

**Authors:** Zheng Yin, Stephen T.C. Wong

**Affiliations:** Department of Systems Medicine and Bioengineering, Houston Methodist Cancer Center and Ting Tsung & Wei Fong Chao Center for BRAIN, Houston Methodist Research Institute, Weill Cornell Medicine, Houston, TX, USA

**Keywords:** Alzheimer’s disease, drug repositioning, modeling, multi-omics, prediction, systems biology, validation

## Abstract

Alzheimer’s disease and related dementias (AD/ADRD) affects more than 50 million people worldwide but there is no clear therapeutic option affordable for the general patient population. Recently, drug repositioning studies featuring collaborations between academic institutes, medical centers, and hospitals are generating novel therapeutics candidates against these devastating diseases and filling in an important area for healthcare that is poorly represented by pharmaceutical companies. Such drug repositioning studies converge expertise from bioinformatics, chemical informatics, medical informatics, artificial intelligence, high throughput and high-content screening and systems biology. They also take advantage of multi-scale, multi-modality datasets, ranging from transcriptomic and proteomic data, electronical medical records, and medical imaging to social media information of patient behaviors and emotions and epidemiology profiles of disease populations, in order to gain comprehensive understanding of disease mechanisms and drug effects. We proposed a recursive drug repositioning paradigm involving the iteration of three processing steps of modeling, prediction, and validation to identify known drugs and bioactive compounds for AD/ADRD. This recursive paradigm has the potential of quickly obtaining a panel of robust novel drug candidates for AD/ADRD and gaining in-depth understanding of disease mechanisms from those repositioned drug candidates, subsequently improving the success rate of predicting novel hits.

Alzheimer’s Disease and Alzheimer’s Disease Related Dementias (AD/ADRD) is the most common forms of dementia, contributing to 60–70% of ∼50 million dementia cases around the world [1]. Approximately 6.2 million Americans age 65 and older are living with AD in 2021. The enormous social and economic burdens created by AD/ADRD have been amplified by the COVID-19 pandemic. In 2020, the deaths among AD/ADRD patients increased by 16% comparing to the average of the previous five years, and the pandemic-related caregiving challenges have caused various negative outcomes among professional caregivers as well as patients’ relatives [2].

The conventional drug discovery paradigms seem ill-equipped to combat a disease as complex as AD. More than 99% of clinical trials for AD therapeutics failed to show significant drug-placebo differences [3]. In June 2021, Aducanumab, an amyloid beta-directed monoclonal antibody, gained FDA approval as a treatment of AD in the US. “The first new treatment approved for AD since 2003” immediately drew mixed responses from the AD community and the public [4]. Its enormous price tag (∼$56,000 per year), relatively complicated administration scheme (through IV injection, once every four weeks), and associate costs of imaging tests via magnetic resonance imaging (MRI) or positron emission tomography (PET) brought concerns on disparity in accessing treatments, as ∼60% AD patients live in low- or middle-income countries [1], and particularly in the US, older Black (18.6% among adults age 65 and older) and Hispanic (14%) Americans are more likely than older Caucasian (10%) Americans to have AD, and non-White racial/ethnic populations expect and experience more barriers when accessing dementia care [2].

It is also under scrutiny that Aducanumab has such straightforward but somehow singular mechanism of action, which is to reduce Aβ oligomers/aggregates and subsequential neuron deaths. While AD/ADRD is characterized by cognitive impairment, neurodegeneration, accumulation of β-amyloid (Aβ), and hyperphosphorylated Tau (pTau) [5]. More and more factors are being linked to the onset and progression of AD, highlighted by neuroinflammation while also including biological processes like insulin resistance [6] and risk factors like heavy metal exposures [7], gut microbiome irregularity [8] and infections [9]. Yet so far such swiftly growing panel of potential therapeutic targets has produced very limited options to halt the disease progression. Routine failures of various novel disease therapies in clinical trials have made the development of anti-AD drugs as an extreme high-risk activity for the pharmaceutical industry.

Drug repositioning is a promising form of drug discovery that aims to use existing drugs, shelved drugs or candidates that failed clinical trials for different diseases. Recently, drug repositioning studies featuring collaborations between academic institutes, medical centers, and hospitals are generating novel therapeutics candidates against devastating diseases, including the work from our own research center on repositioning drugs for groups 3–4 medulloblastoma [10], Ewing Sarcoma [11], and metastatic breast cancer [12], as well as filling gaps of healthcare that is poorly represented by pharmaceutical companies, e.g. controlling chemotherapy induced diarrhea [13] and alleviating cancer drug toxicity [14]. These drug repositioning studies converge expertise from bioinformatics, chemical informatics, medical informatics, systems biology, high throughput and high-content screening, they also take advantage of multi-scale, multi-modality datasets, from transcriptomic and proteomics profiles, electronical medical records and medical imaging to social media and epidemiology surveillance, in order to gain comprehensive understanding of disease mechanisms and drug effects. It is the genuine hope of the AD/ADRD community that such systematic drug repositioning methods could deliver novel therapeutics options for AD, first in the form of economic and effective alternatives for aducanumab, and later leveraging the knowledge gained on AD/ADRD disease mechanism to discover new drugs. To support such hope, National Institute of Aging (NIA) established the program for “Translational Bioinformatics Approaches to Advance Drug Repositioning and Combination Therapy Development for Alzheimer’s Disease,” which is a part of the efforts towards the National Alzheimer’s Project Act (NAPA) goal of effectively treating or preventing AD/ADRD by 2025.

Given the complexity of AD/ADRD pathogenesis and associated co-morbid conditions, both the “depth” and “width” of currently drug repurposing solutions need to be improved in order to deliver effective AD therapeutic solutions. The depth of a drug-repurposing project refers to the level of understanding on disease mechanism and drug-target interactions. Improving depth requires a reliable AD model system, which recapitulates AD pathogenesis in a human brain-like environment, and in-depth transcriptomic profiles, which can reveal molecular-level changes underlying disease-reversing phenotypes. On the other hand, the width of a therapeutics search relies on the efficacy of predicting and validating effects of candidate compounds from an enormous search space. Width can be gained from novel computational algorithms connecting – omics changes with phenotypic changes, thus guiding the search with enriched knowledge on mechanisms and avoiding exhaustive, costly, and time-consuming testing of every available drug.

We propose an effective drug repositioning paradigm for AD/ADRD by recursively looping the processing steps of modeling, prediction, and validation, see Figure 1.

**Figure 1:**
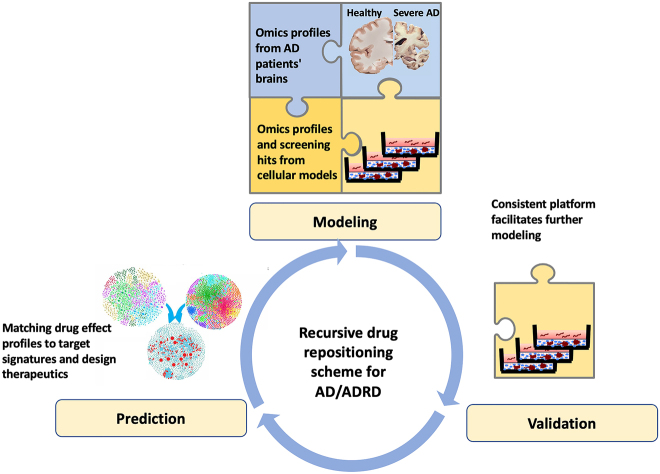
A recursive “modeling → prediction → validation” paradigm for AD/ADRD drug repositioning.

The modeling step transfers relevant disease phenotypes of AD/ADRD into itemized target signatures to be used for drug development. It can be achieved by dissecting imaging and multiple-omics data from AD/ADRD patients, but it will be beneficial if the data used for prediction and validation is generated from similar model systems used to establish the target signature. There are increasing use of 3D cellular systems or animal models like Drosophila to initiate AD/ADRD drug repositioning. Comparing to the natural progression of AD in patients over decades or in transgenic mouse models over 8–10 months, cellular models can capture key AD pathology like accumulation of pTau within 8–12 weeks [15].

Next, the prediction step matches the itemized target signature to multi-omics profiles on drug effects. A popular hypothesis searches for compounds that can reverse the omics changes identified from disease signatures; alternatively, domain knowledge or small-scale screenings can generate primary hit compounds (e.g., aducanumab in Aβ modeling systems for oligomers/aggregates) and the prediction step focuses on finding compounds with similar impact on multiple omics and better pharmaceutical characteristics. In both cases, changes in multiple omics profiles may not be related to AD or not able to be directly impacted by compounds. Genetics and chemical informatics analysis are needed to prioritize the changes achievable by drugs, and combinations of drugs with favorable pharmaceutical features or drugs each covering part of the target signatures need to be considered and designed.

Then, the validation step applied predicted candidates on AD model systems to test their efficacy. While the primary screening may use imaging to identify candidates reducing/clearing pTau, enzyme-linked immunosorbent assay (ELISA) is available for the secondary screening to quantify the candidates’ impact on pTau and record their effects on soluble and insoluble Aβ pathology involving different isoforms. Such comprehensive biochemistry quantifications allow the researchers to leverage the validation results to carry out a new round of modeling, update the target signatures and initiate the next round of prediction with the AI based drug repositioning screening technology guided by more reliable target signatures obtained from the validations. Meanwhile, the validated hits would enter pre-clinical validations for their beneficial effects on reversing cognitive impairments. Successful pre-clinical validations request (1) appropriate animal model with compatible AD pathology factors as the screening platform; (2) focusing on behavioral tasks echoing defects in humans affected by AD and related to signaling pathways manifested in the screening model, e.g. Y-maze test studies short-term memories and is shown to be mediated by interferon signaling; and (3) comprehensive neuropathology test for factors that changes with drug treatment, e.g. neuroinflammation, amyloid load, neuritic tau pathology, synaptic integrity and neuronal loss.

The proposed framework requests integration of multi-modality datasets, and Table 1 summarized several publicly available datasets which may contribute to the modeling, prediction and/or validation steps of AD drug repositioning.

**Table 1:** Examples of publicly available datasets for AD drug repositioning.

Database/website	Dataset/tool description	Data type	Potential usage
https://adknowledgeportal.synapse.org	The Religious Orders and Memory and Aging project (ROS/MAP)	Multi-Omics	Modeling
	The Mount Sinai Brain Bank Study (MSBB)		
	The RNAseq Harmonization study for uniformly processed RNAseq dataset across all AMP-AD studies	RNAseq	
Clue.io	Broad Institute LINCS dataset	Transcriptomics from L1000 arrays	Prediction
	Touchstone signature mapping	Similarity scores	
http://www.swissdock.ch	Protein-ligand interaction prediction	Possibility scores	
https://dockthor.lncc.br/v2/			
https://dailymed.nlm.nih.gov/dailymed/	Side effect information for approved drugs	Text	Validation
http://sideeffects.embl.de			

Although fluorescent imaging (for screening and validation) and transcriptomic profiles (for modeling AD pathology and predicting candidates) are major data types supporting the proposed framework, other data modalities can be incorporated into the workflow, e.g. (1) Genetic profiles, medical records on mental cognitive status, white matter hyperintensities (WMH) obtained through *in vivo* MRI imaging and post-mortem imaging-based scores on AD related pathologies have been used to accurately define patient population used for modeling and generating target signatures; (2) single cell RNAseq data is being utilized to model the defect of neuron-glia and glia-glia communications manifested in AD brain microenvironment; (3) molecular structures for target proteins and small-molecule compounds can help predict significant protein-drug binding process underlying favorable phenotypes; (4) pharmaceutical profiles for candidate compounds, e.g. toxicity, ability to cross blood–brain barrier etc. help identify practical target for further validations; (5) multi-modality imaging and -omics profiles are recruited to the validation process in order to accurately quantify the beneficial effects of the candidates. The recursive paradigm and the consistency between modeling systems used for generating of target signature and validating the candidates help circumvent a major challenge facing the prediction process, i.e., the lack of multi-omic compound effect profiles in AD models. Popular publicly available transcriptomic databases for compound effects, e.g., Clue.io hosted by the Broad Institute, contain limited data on neural cells. Establishing a multi-omic database focusing on neurons and glia cells in AD brain microenvironment allows all three steps of modeling, prediction, and validation to happen in the same model system and converges multi-modality datasets for drug repositioning in AD/ADRD.
